# Design and Main Results of STURDY: A Randomized Clinical Trial of Four Vitamin D3 Doses to Prevent Falls in Older Adults

**DOI:** 10.1093/geroni/igaa057.2737

**Published:** 2020-12-16

**Authors:** Lawrence Appel, Jennifer Schrack, Erin Michos, Christine Mitchell, Stephen Juraschek, Edgar Miller, David Roth

**Affiliations:** 1 Johns Hopkins University School of Medicine, Baltimore, Maryland, United States; 2 Johns Hopkins Bloomberg School of Public Health, Baltimore, Maryland, United States; 3 Johns Hopkins School of Medicine, Baltimore, Maryland, United States; 4 Harvard Medical School, Boston, Massachusetts, United States; 5 Johns Hopkins University, Baltimore, Maryland, United States

## Abstract

STURDY was a Bayesian, response-adaptive trial with dose-finding and confirmatory stages. Participants (n=688; ≥70years with serum 25(OH)D of 10-29ng/mL) were randomized to 200 (control), 1000, 2000, or 4000 IU/day of vitamin D3. The primary outcome was time to first fall or death over 2 years. During dose-finding, the best non-control dose was determined to be 1000IU/day based on higher primary outcome event rates in the 2000 and 4000IU/day doses than the 1000IU/day dose (posterior probability of being best dose=0.90; hazard ratios[HR] were 1.86 [95%CI: 1.16-2.97] and 1.68 [95%CI: 1.05-2.69], respectively). Participants were then switched from other non-control doses to 1000IU/day, and event rates did not differ between the pooled higher doses and control groups (HR=1.02, P=0.84). There was no heterogeneity by baseline 25(OHD). In conclusion, high-dose vitamin D supplementation ≥1000IU/day did not prevent falls. Whether vitamin D doses >2000IU/day increase the risk of falls is uncertain.

